# A Selective Ratiometric Fluorescent Probe for No-Wash Detection of PVC Microplastic

**DOI:** 10.3390/polym13101588

**Published:** 2021-05-14

**Authors:** Valeria Caponetti, Alexandra Mavridi-Printezi, Matteo Cingolani, Enrico Rampazzo, Damiano Genovese, Luca Prodi, Daniele Fabbri, Marco Montalti

**Affiliations:** 1 Department of Chemistry “Giacomo Ciamician”, University of Bologna, Via Selmi 2, 40126 Bologna, Italy; valeria.caponetti2@unibo.it (V.C.); alexandra.mavridi2@unibo.it (A.M.-P.); matteo.cingolani4@unibo.it (M.C.); enrico.rampazzo@unibo.it (E.R.); damiano.genovese2@unibo.it (D.G.); luca.prodi@unibo.it (L.P.); dani.fabbri@unibo.it (D.F.); 2 Tecnopolo di Rimini, Via Dario Campana, 71, 47922 Rimini, Italy

**Keywords:** microplastic, ratiometric detection, no-wash fluorescent probe, imaging, one-pot reaction, water remediation, nanoplastic

## Abstract

Microplastics (MP) are micrometric plastic particles present in drinking water, food and the environment that constitute an emerging pollutant and pose a menace to human health. Novel methods for the fast detection of these new contaminants are needed. Fluorescence-based detection exploits the use of specific probes to label the MP particles. This method can be environmentally friendly, low-cost, easily scalable but also very sensitive and specific. Here, we present the synthesis and application of a new probe based on perylene-diimide (PDI), which can be prepared in a few minutes by a one-pot reaction using a conventional microwave oven and can be used for the direct detection of MP in water without any further treatment of the sample. The green fluorescence is strongly quenched in water at neutral pH because of the formation dimers. The ability of the probe to label MP was tested for polyvinyl chloride (PVC), polyethylene (PE), polyethylene terephthalate (PET), polypropylene (PP), polystyrene (PS), poly methyl methacrylate (PMMA) and polytetrafluoroethylene (PTFE). The probe showed considerable selectivity to PVC MP, which presented an intense red emission after staining. Interestingly, the fluorescence of the MP after labeling could be detected, under excitation with a blue diode, with a conventional CMOS color camera. Good selectivity was achieved analyzing the red to green fluorescence intensity ratio. UV–Vis absorption, steady-state and time-resolved fluorescence spectroscopy, fluorescence anisotropy, fluorescence wide-field and confocal laser scanning microscopy allowed elucidating the mechanism of the staining in detail.

## 1. Introduction

In the last years, due to the rising demand of plastic items, plastics that are considered as the greatest invention of the last century, are now one of the main threats for both marine and terrestrial ecosystems [1]. In particular, microplastics (MP), which are plastic particles of micrometric size, are considered emerging contaminants as MP debris are abundantly found in many seas and rivers [2,3,4], but also in urban environments, reaching even high-level organisms as humans through the food chain due to bioaccumulation [5]. The accumulation of MP in the aquatic environment leads to physical, but also chemical, changes to the ecosystem, as, according to studies, hydrophobic MP are able to concentrate, via partitioning, and eventually re-release organic pollutants [6]. Acting as “Trojan horses”, they transport faster and more efficiently the pollutants inside the cells promoting also the bioaccumulation of these organic contaminants in many living organisms such as fishes, crustaceans and mollusks, as well as insects, where their toxicological effects have been demonstrated [5,7]. For example, MP have been reported to affect the homeostasis of dragonfly larvae and zebrafish, causing also REDOX imbalance and increased immunity [8,9].

Indeed, MP are estimated to be more than 50% of the field-retrieved plastic debris found in organisms of all kinds and can be classified as primary and secondary depending on their origin [10]. Primary MP are intentionally manufactured micron-sized plastics, whereas secondary MP are derived by the fragmentation of larger plastic products [11,12]. The main types of MP debris found in the environment are polyethylene (PE), polypropylene (PP), polystyrene (PS), polyvinyl chloride (PVC) and polyethylene terephthalate (PET). Among them, PVC, due to its high density, more easily settles in freshwaters, which in many cases are drinking water sources, being less available during environmental sampling but more available to benthonic organisms [13]. Thus, it can be hypothesized that the real percentage of this polymer in the marine environments is probably underestimated, increasing the need for its detection. Moreover, PVC MP pose a very serious threat to the human organism, as they can circulate in the body and their long exposure can cause immunotoxicity to the cells stimulating the release of interleukin 6 (IL-6) and tumor necrosis factor-α (TNF-α) [14,15]. Large concentrations of PVC can affect freshwater photosynthetic systems, e.g., *Chlorella pyrenoidosa* and *Microcystis flos-aquae algae*, by inhibiting the photosynthetic ability and growth of the organisms [16]. Similar detrimental effects of PVC MP have also been reported in the case of marine phytoplankton, causing dose-dependent toxicity in various diatoms [17]. Thus, it is obvious that methods for the detection of PVC MP in environmental samples, biological tissues or even consumer products are urgently needed to determine the occurrence of this MP in the world.

**Figure 1:**
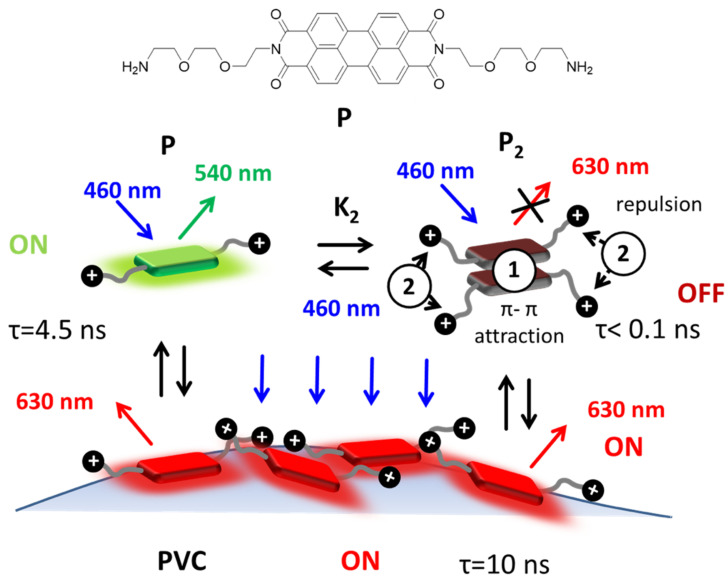
Structure of the fluorescent probe P that includes a central hydrophobic perylene-diimide (PDI) unit and two hydrophilic amino-terminated branches that are protonated in water at pH 7. In neutral water solution, P is, to a large extent, aggregated in the form of very weakly red emitting dimer, P_2_, with very short excited state lifetime τ < 0.1 ns. Upon adsorption on polyvinyl chloride (PVC), P becomes intensely red fluorescent and the excited state lifetime increases to 10 ns. The attractive π–π and repulsive electrostatic interactions between P molecules are schematized as 1 and 2. Adsorption of PVC leads to disaggregation and switch-on the red fluorescence (this mechanism is discussed in detail in the main text).

The detection of MP can be achieved with the use of fluorescent probes [18,19] able to interact and, therefore, stain the polymers. In most cases, Nile Red, able to bind to different polymers leading to colors from yellow to red depending on the plastic’s hydrophilicity, has been exploited as a fluorescent probe for the visual inspection and categorization of different MP [20,21,22,23,24]. Nile Red derivatives [25,26] have been presented as possible candidates able to improve the water solubility and selectivity of Nile Red [27]. Nevertheless, there are very few examples of other fluorescent probes. Some examples are the use of commercially available iDyes [28], Rhodamine B [29] and pyrene [30,31], all working optimally in organic solvents or requiring time consuming techniques. Perylene diimides (PDIs) are a class of industrial organic dyes which have been applied for a wide range of applications due to their stability and outstanding optical and electronic properties [32,33,34]. Focusing on the great need for PVC MP screening, we propose a highly selective, simple and time-saving approach based on a perylene diimide probe (P) shown in Figure 1, which can be carried out in water. This method has the potential to become an outstanding monitoring tool due to its low cost and high throughput.

For the detection of MP in real samples at a global level, fluorescent probes should be easily producible in large scale with environmentally friendly and inexpensive processes. Thus, we propose the use of microwave irradiation to achieve gram-scale production of the probe in a few minutes using a conventional microwave oven.

The main objective of this study was to exploit the effect of aggregation on the fluorescence of PDIs to achieve a no-wash selective probe for MP. In particular, the probe P was designed to be soluble in water at neutral pH, where it can form quenched aggregates giving no background signal and become fluorescent upon absorption on MP. As a result, the fluorescence of the MP can be detected directly in the staining solution without any washing or further treatment.

## 2. Materials and Methods

All reagents, solvents and chemicals were purchased from Sigma-Aldrich (Steinheim, Germany) and used without modification, unless otherwise stated. In particular, the following polymers were purchased (the number in brackets is the Sigma-Aldrich catalogue code): polyvinyl chloride (PVC, 346764) with average Mw = 233,000, medium density polyethylene (PE, 332119), isotactic poly methyl methacrylate (PMMA, 452130), polytetrafluoroethylene (PTFE, 430935), isotactic polypropylene (PP, 428116) with average Mw = 12,000, polyethylene terephthalate (PET, 429252) and polystyrene (PS, 327743) with average Mw 20,000. Note that PP and PET were ground with commercial lab grinder prior to use; their final average grain sizes were 600 ± 300 and 400 ± 200 µm, respectively. Polyaniline (530689) with average Mw = 65,000, poly(acrylic acid) (323667) with average Mw = 1800, poly(acrylic acid) (181285) with average Mw = 450,000, poly(styrene sulfonic acid sodium salt) (81612) with average Mw = 77,000, poly(styrene sulfonic acid sodium salt) (81615) with average Mw = 350,000 and poly(vinyl alcohol) with average Mw = 130,000 were also tested.

*Photophysical characterization*. The experiments were carried out in air-equilibrated solutions at 25 °C. UV–Vis absorption spectra were recorded with a Perkin-Elmer Lambda 650 (Boston, MA, USA) or Perkin-Elmer Lambda 45 spectrophotometer (Boston, MA, USA) using quartz cells (Hellma, Müllheim, Germany) with path length of 1.0 cm or 2.5 mL macro PMMA or UV disposable cuvettes purchased from BRAND (Wertheim, Germany). The fluorescence spectra were recorded with a Horiba Jobin Yvon Fluoromax-4 (Kyoto, Japan), a Perkin-Elmer LS-55 or an Edinburgh FLS920 (Livingston, UK) equipped with a photomultiplier Hamamatsu R928 phototube (Hamamatsu, Japan). The same instrument connected to a TCC900 card was used for Time Correlated Single Photon Counting (TCSPC) experiments with an LDH-P-C 405 or 635 pulsed diode laser. The fluorescence quantum yields (uncertainty, ±15%) were determined using fluorescein solution in water as a reference with Φ = 1.0 or [Ru(bpy)_3_]^2+^ in water with Φ = 0.028 [35]. The emission intensities were corrected taking into consideration the inner filter effect according to the standard methods [35]. For the fluorescence anisotropy measurements, an Edinburgh FLS920 equipped with Glan-Thompson polarizers (Livingston, UK) was used. The data were corrected for polarization errors using the G-factor.

*Fluorescence microscopy.* Polystyrene 24 multi-well costar 3526 plates were used for the preparation of the samples. The set up used for images acquisition was made by an Olympus IX 71 inverted microscope (Shinjuku, Japan) equipped with a Blue diode (470 ± 20 nm, LZ1-10DB00, Led-Engin, Wilmington, MA, USA) for fluorescence excitation and a Basler acA5472-17uc camera with a Sony IMX183 CMOS sensor. The irradiation blue diode was focused on the back focal plane of the objective (4× magnification, Olympus UPLFLN 4X), with a 50 mm focal length after filtering with a band pass filter (469 ± 17.5 nm, Thorlabs, Newton, NJ, USA) and after 90° reflection on a dichroic filter (452–490 nm/505–800 nm, Thorlabs, Newton, NJ, USA). Fluorescence was filtered with a cut-off emission filter (50% transmission at 510 nm, Thorlabs, Newton, NJ, USA).

*Confocal microscope.* A Nikon A1 (Tokyo, Japan) equipped with a module for fluorescence lifetime image (FLIM) by Picoquant with a pulsed excitation laser at 405 nm with a pulse width of ~70 ps was used.

*Dimeric* vs. *isodesmic model for the aggregation*.

The equilibrium for the formation of the dimer P_2_ from P is [36]:

$$P+P⇄P_{2}$$

Denoting [P] and [P_2_] as the equilibrium molar concentrations of P and P_2_, respectively, the equilibrium constant K_2_ is:

$$K_{2}=\frac{\left[P_{2}\right]}{{\left[P\right]}^{2}}$$

Hence, the molar fraction of monomer, $\chi_{P}=\left[P\right]/C_{P}$, with $C_{P}$ the total concentration of P, can be expressed as:

$$\chi_{P}=\frac{-1+\sqrt{1+8K_{2}C_{P}}}{4K_{2}C_{P}}$$

In the case of the formation of larger aggregates (trimer P_3_, tetramer P_4_, etc.), additional equilibria have to be considered:

$$P+P⇄P_{2};{\;P}_{2}+P⇄P_{3};\;P_{3}+P⇄P_{4};\ldots$$

Other association constants, K_3_ for the trimer P_3_, K_4_ for the tetramer P_4_, etc., have to be considered:

$$K_{3}=\frac{\left[P_{3}\right]}{\left[P_{2}\right]\left[P\right]};{\;K}_{4}=\frac{\left[P_{4}\right]}{\left[P_{3}\right]\left[P\right]};\ldots$$

A simple model for the aggregation is the isodesmic one, which considers:

$$K=K_{2}=K_{3}=K_{4},\ldots$$

In this case, a different dependency of the molar fraction of the monomer on the total concentration of P is expected according to the following equation:

$$\chi_{P}=\frac{2{\mathrm{KC}}_{P}+1-\sqrt{4{\mathrm{KC}}_{P}+1}}{2K^{2}C_{P}^{2}}$$

### 2.1. Preparation and Purification of P Probe

For the synthesis of P (N,N′-Bis(Ethyl-diethoxyethyl-ethylamine)perylene-3,4,9,10-bis(dicarboximide), 200 μL of 2,2-(Ethylenedioxy)bis(ethylamine) (1 mmol) were mixed in 10 mL ethylene glycol in a 100 mL Erlenmeyer flask [37,38]. Afterwards, 20 mg (0.05 mmol) of perylene-3,4,10-tetra-carbocyl dianhydride (PDA) were added to the previous, and the mixture was sonicated for 1 min. The reaction was carried out in a commercial microwave oven (2.45 GHz, 900 W, Benton Harbor, Whirlpool, MI, USA) to achieve the conversion of PDA to PDI-probe, P in 5 min. A color change was observed from red to purple. The product was then precipitated by adding 60 mL of water and isolated by centrifugation at 5000 rpm for 10 min and washed three times with distilled water. The final product was deep purple, and it was dried under vacuum for 24 h.

### 2.2. Characterization of P

^1^H-NMR spectra were recorded on Varian 400 (400 MHz) spectrometers (Palo Alto, CA, USA). Chemical shifts are reported in ppm from TMS with the solvent resonance as the internal standard (deuterochloroform: 7.24 ppm). ^13^C-NMR spectra were recorded on a Varian 400 (100 MHz) spectrometers with complete proton decoupling. Chemical shifts are reported in ppm from TMS with the solvent as the internal standard (deuterochloroform: 77.0 ppm).

^1^H NMR (400 MHz, DMSO-d6, acetic acid-d4) δ (ppm): 2.97 (t (broad), 4H,), 3.38–3.70 (m (broad), 20 H,), 4.20 (s (broad), 4 H), 8.00–8.32 (m (broad), 8 H)

^13^C NMR (75.5 MHz, DMSO-d6, acetic acid-d4) δ (ppm): 38.5, 50.8, 59.0, 66.8, 69.6, 69.8, 118.1, 118.7, 124.0, 125.7, 132.8, 138.8, 165.0.

Electrospray ionization (ESI) mass spectra were obtained with Agilent Technologies MSD1100 single-quadrupole mass spectrometer (Santa Clara, CA, USA). Mass spectra were acquired in full-scan mode from m/z 150 to 3000, with a scan time of 0.1 s in the positive ion mode. The parameters of the spray chamber were set as follows: drying gas flow at 10 mL min^−1^, nebulizer (nitrogen) pressure at 35 psig, temperature at 350 °C and ESI spray voltage at 5000 V. The fragmentor voltage was maintained at 50 V.

Chromatographic analysis with HPLC-MS (Agilent Technologies, Santa Clara, CA, USA) showed a single peak, proving the purity of the product. The masses detected were m/z = 653.4 and m/z = 327.3, corresponding to PH^+^ (expected m/z = 653.3) and PH_2_^+^ (expected m/z = 327.2), respectively.

High-resolution MS (HRMS) ESI analyses were performed on a Xevo G2-XS QTof mass spectrometer (Waters, Milford, MA, USA). Mass spectrometric detection was performed in the full-scan mode from m/z 50 to 1200, with a scan time of 0.15 s in the positive ion mode and the following settings: cone voltage of 40 V and collision energy of 6.00 eV. ESI was performed with the settings: capillary of 3 kV, cone of 40 V, source temperature of 120 °C, desolvation temperature of 600 °C, cone gas flow of 50 L/h and desolvation gas flow of 1000 L/h.

The exact mass was m/z = 653.259 (expected for PH^+^ m/z = 652.253).

### 2.3. Method for Detection of the MP

A water-buffered solution of P (c = 8 µM, phosphate buffer pH = 7) was prepared for the staining of the MP. In particular, PVC, PE, PET, PP, PS, PMMA and PTFE were tested. For the staining, 5 mg of polymer were placed in a 24-well plate and incubated for 2 h with 2 mL of the above-described P-buffered solution. In the next step, the fluorescence images of the polymers were acquired directly in the solution with the fluorescence inverted microscope described above.

## 3. Results and Discussion

This section describes and discusses the applicability of the probe P for the direct staining of MP in neutral water making them fluorescent and, therefore, easily detectable with a conventional fluorescence microscope. More in detail, two main advantages of this approach are pointed out: (i) the probe P aggregates in neutral-buffered water solution giving almost completely quenched dimers; and (ii) P binds selectively to PVC microplastic, giving a characteristic red fluorescence. As a consequence, it is possible to selectively detect PVC MP by simply mixing the probe in a water suspension of the MP and imaging the suspension without any washing or further treatment, since the fluorescence signal from the background is very weak.

An essential prerequisite of this approach is the use of a water-soluble probe, which still presents a high tendency to aggregate, forming non-fluorescent species. For this reason, we designed the new probe molecule P by functionalizing the fluorophore with two hydrophilic di-ethylene glycolic chains terminated with amino groups that are protonated at neutral pH (pKa = 9.04 ± 0.10), increasing the hydrophilicity of the molecule. These short and linear chains confer to the molecule the required solubility in water, but they are not bulky enough to prevent the aggregation and quenching of the fluorescence. Additionally, the absence of any bulky group directly bound to the chromophore makes possible the interaction of the hydrophobic PDI with the surface of the polymers and, in particular, PVC.

Before testing the ability of P to stain the various MP, we investigated its photophysical properties by UV–Vis absorption spectroscopy and steady-state and time-resolved fluorescence spectroscopy. PDI derivatives are known to aggregate, especially in polar environment, because of strong π–π stacking interactions involving the extended aromatic chromophores. Depending on the solvent and the molecular structure, in particular the presence of bulky substituents, the aggregation can lead to the formation of dimers or oligomers or to formation of large aggregates, up to large nanoaggregates. In these aggregates, the photophysical properties of PDI are widely modified with respect to the starting monomer [32,33,34].

**Figure 2:**
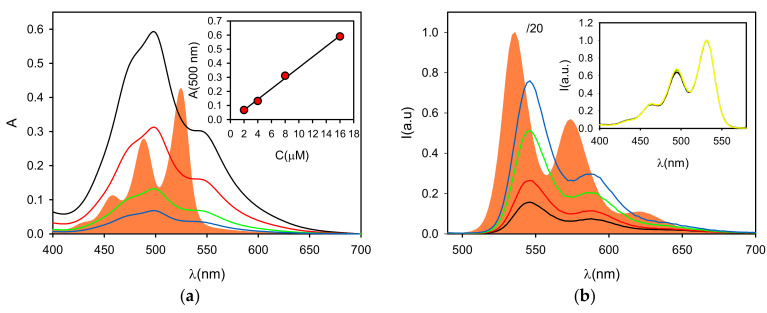
(**a**) Absorption spectra of P in DCM (filled orange, C = 5.3 µM) and of P in buffered neutral water (pH = 7) at concentrations 16, 8, 4 and 2 µM (black, red, green and blue lines, respectively). The absorbance at 500 nm as a function of the concentration is plotted in the inset. (**b**) Fluorescence spectra (λ_exc_ = 470 nm) of P in DCM (filled orange, C = 5.3 µM, divided by 20 for scaling) and P in buffered neutral water (pH = 7) at concentrations 16, 8, 4 and 2 µM (black, red, green and blue lines, respectively). The normalized excitation spectra for the four water solutions (λ_em_ = 580 nm) are shown in the inset (same color line). All fluorescence and excitation spectra are corrected for the inner filter effect.

To investigate the effect of the aggregation of P in water, we first characterized the molecule in dichloromethane (DCM). In fact, for similar molecules, it has been reported that aggregation in this solvent is purely efficient, which allows investigating the properties of PDI in the non-aggregated form [33].The UV–Vis absorption spectrum of P (5.3 µM) in DCM is shown in Figure 2. As shown in the figure, the spectrum exhibited the vibrationally structured bands, typical of the PDI monomer, with a maximum at λ_max_ = 525 nm and a molar absorption coefficient ε = 8 × 10^4^ M^−1^ cm^−1^. It should be stressed that the presence of the vibrational peaks for the electronic transition (S_0_–S_1_) in the 400–550 nm range is typical of the monomeric form of PDI, while aggregates (typically H-type) present a much less structured band [32,36].

The fluorescence spectrum (λ_exc_ = 480 nm) in DCM, as illustrated in Figure 2, showed a small Stoke shift with respect to the absorption one, and it appeared as the mirror image of the absorption spectrum, maintaining the typical vibrational spectral structure, as also reported for other PDI-based fluorophores. More in detail, the maximum is positioned at λ_max_ = 535 nm, and the fluorescence quantum yield was found to be Φ = 40%, which is lower than reported for similar PDI molecules (~100%) [36]. The excitation spectrum of P in DCM (λ_em_ = 480 nm, not reported) perfectly matched the absorption spectrum. The partial quenching of the fluorescence was confirmed by time resolved fluorescence analysis and in particular by TCSPC that allowed recording a deactivation kinetic of the excited state. The latter could be fitted with a mono-exponential model to give a lifetime of τ = 3.2 ns, while, for similar PDI monomers, a singlet excited state lifetime τ = 3.9 ns has been reported [39,40].

The decrease of the fluorescence quantum yield and of the excited state lifetime of P, with respect to analogous molecules, could be attributed to the presence of the two terminal amino groups which are not protonated in DCM. In fact, amines are known to dynamically quench excited state fluorescence, via electron-transfer process, producing the reduced PDI and the oxidized amine; this process has been widely explored in organic synthetic photocatalysis [41,42]. In the case of dynamic (diffusional) quenching, the back-diffusion of the encounter pair and/or the irreversible degradation of the oxidized amine can lead to the stabilization of the anionic PDI, at least in the absence of oxygen [41]. On the other hand, in the case of P, the quenching is static, and the charge-separated state undergoes fast recombination, producing a partial quenching of the fluorescence. This mechanism was demonstrated by adding trifluoroacetic acid (20 µM) to the P solution in DCM in order to protonate the amines and therefore produce ammonium species which cannot be oxidized by the excited PDI. Indeed, upon protonation, a considerable increase in the lifetime was observed (τ = 4.3 ns).

In the next step, the photophysical behavior of P was investigated in water buffered (pH = 7, phosphate buffer) solution. In particular, since aggregation of P was expected to be strongly concentration-dependent, we prepared a set of different concentrations of the probe in this solvent (0.2 × 10^−5^, 0.4 × 10^−5^, 0.8 × 10^−5^ and 1.6 × 10^−5^ M, referred to as solutions P1, P2, P3 and P4, respectively). The UV–Vis absorption spectra of these water solutions of P are reported in Figure 2. They exhibited broader absorption bands in neutral water with poorly-defined vibrational structures and a maximum at λ_max_ = 498 nm. These spectral changes are characteristic of the H-aggregates formed by PDI dyes that tend to self-assemble and form π–π stacked species, especially in protic solvents such as water.

Since the spectral signature is, to some extent, characteristic for the kind of aggregation and it reflects the ratio between aggregated and non-aggregated molecules, the absorbance spectra after normalization were compared to distinguish any effect of the concentration. All spectra were very well superimposed, and, as shown in Figure 2, the absorbance at 498 nm was found to be directly proportional to the concentration. As a consequence, the molar absorption coefficients, as shown in Table 1, were widely concentration independent. On the other hand, the fluorescence spectra, upon 470 nm excitation, confirmed the presence of a very minor fraction of the free monomer (in all cases, <2%). In fact, looking at the fluorescence spectra shown in Figure 2, the emission profile did not significantly change in water, possessing an emission maximum at 546 nm and the typical structured green fluorescence band of the PBI monomer. The origin of this green emission was confirmed by the excitation spectra of the water solutions at λ_em_ = 580 nm shown in Figure 2, which again presented the typical features of the P monomer, and by the excited state lifetime at λ_em_ = 540 nm.

In fact, the decay of the excited state in neutral water was mono-exponential and the excited state lifetime remained almost unaltered upon concentration change. As shown in Table 1, it was found to be τ ~4.5 ns, which is similar to the value observed for P in DCM after protonation. Further information about the origin of the green fluorescence band was given by fluorescence anisotropy spectroscopy. This technique was chosen for the analysis of the reorientation of the excited state dipole, after excitation, during deactivation. The value of fluorescence anisotropy, r, measured for the green fluorescence was quite low, about 0.03 at all concentrations. This value is compatible with the rotation of monomeric species as P [33].

**Figure 3:**
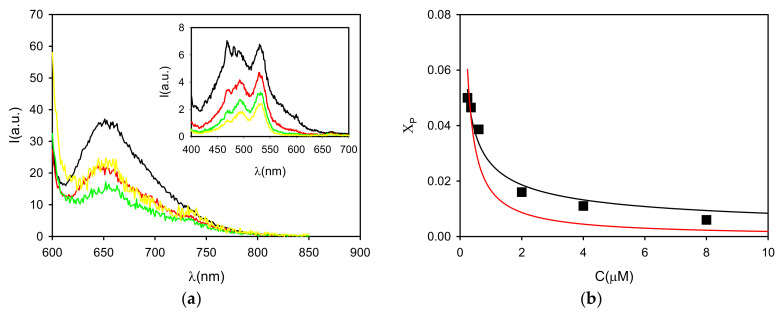
(**a**) Fluorescence spectra of P in buffered neutral water (pH = 7) at concentrations 16, 8, 4 and 2 µM upon λ_exc_ = 590 nm (black, red, green and yellow lines, respectively). The excitation spectra of the four solutions (λ_em_ = 720 nm) are shown in the inset (same color line). (**b**) The fluorescence quantum yield is used to calculate the molar fraction of P (χ_P_) and it is plotted as a function of concentration (black squares). These data are fitted using either a dimeric (black line K_2_ = 7 × 10^8^ M^−1^) or an isodesmic model (red line, K = 5 × 10^7^ M^−1^).

Since the absorption spectra clearly revealed the predominant presence of PDI aggregates, we investigated the fluorescence properties of the P solution upon selective excitation at 590 nm (Figure 3), a wavelength at which the monomer cannot be excited (Figure 2). In this excitation condition, all the four P water solutions presented a weak broad emission at 650 nm, typical for PDI aggregates and excimers. As shown in Table 1, for this emission, the fluorescence quantum yields were extremely weak and the excited state lifetimes very short (<0.1 ns). Different from what was reported for the green fluorescence anisotropy, the one for the red emission was very high (r ~ 0.25), considering that the maximum value possible for a randomly oriented system is r = 0.4. Such a high anisotropy is compatible with the presence of aggregates with higher molar mass with respect to the monomer as well as the short excited-state lifetime.

It must be underlined that, based on their extremely low fluorescence quantum yield, the aggregates can be considered completely quenched with respect to the monomer. Moreover, upon excitation at 470 nm, it is possible to assume that only the fraction of light absorbed by the monomer produces detectable fluorescence. Hence, the average quantum yield in this excitation condition can be used to find the molar fraction of the free monomer. More specifically, as shown in Table 1, the average fluorescence quantum yield in phosphate buffer was found to be much lower (Φ < 2%) than the one measured in DCM, and it was decreasing upon increasing P concentration.

The fraction of free monomer, calculated from the fluorescence quantum yield Φ_1_, was plotted as a function of P concentration (Figure 3) to investigate the process of aggregation. In particular, the experimental data were fitted with both dimeric and isodesmic models. As shown in Figure 3, while the dimeric model allowed fitting the experimental points with K_2_ = 7 × 10^8^ M^−1^, an optimal fitting with an isodesmic model (K = 5 × 10^7^ M^−1^) failed in describing the experimental results. Thus, we concluded that the aggregation leads to the formation of oligomer and mostly P dimer, but not extended structures.

**Figure 4:**
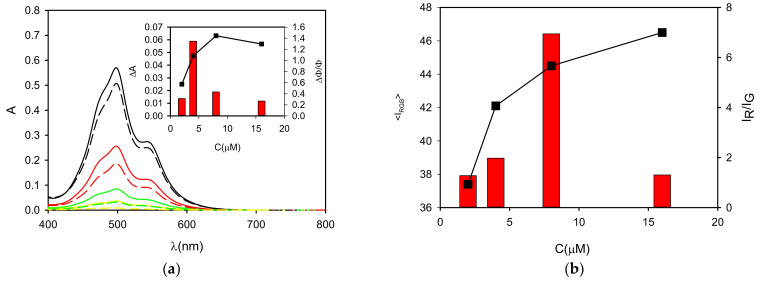
(**a**) Absorption spectra of P in buffered neutral water (pH = 7) at concentrations 16, 8, 4 and 2 µM (black, red, green and yellow lines, respectively) before (continuous line) and after (dashed lines) 2 h incubation with PVC (5 mg in 2 mL solution). The decrease of the absorbance of the solution is plotted in the inset (black squares) as a function of the concentration. The relative increase of the fluorescence intensity of the solution at 540 nm after incubation is plotted as red bars. (**b**) The total intensity measured by wide field microscopy for the PVC particles after the incubation is shown as a function of P concentration (<I_RGB_>). The ratio of the intensity on the red and green channels (I_R_/I_G_) is plotted as red bars.

In the next step, the possibility of P to be applied for the staining of MP was investigated. In particular, the ability of P to selectively stain PVC MP at neutral pH for easy and fast detection of the specific MP debris was verified. Five milligrams of PVC were added to 2 mL of P1–P4 solutions, and they were mixed for 2 h, after which the UV–Vis absorption, fluorescence emission and excitation spectra, as well as excited state lifetime, were acquired again. The absorption spectra of the P1–P4 solutions after 2 h incubation with PVC showed a relevant decrease of the peak at 500 nm without any important change in the spectral profile. On the contrary, from the fluorescence analysis, only a small increase of the average quantum yield of the monomeric (green) fluorescence of P in solution was observed, as shown in Figure 4. These results suggest that the adsorption of P on the PVC surface does not substantially alter the equilibrium of aggregation of P in solution. As before, the interaction of P with PVC was confirmed by excitation spectra, excited state lifetime and fluorescence anisotropy, attributing the remaining green fluorescence to the monomer.

**Figure 5:**
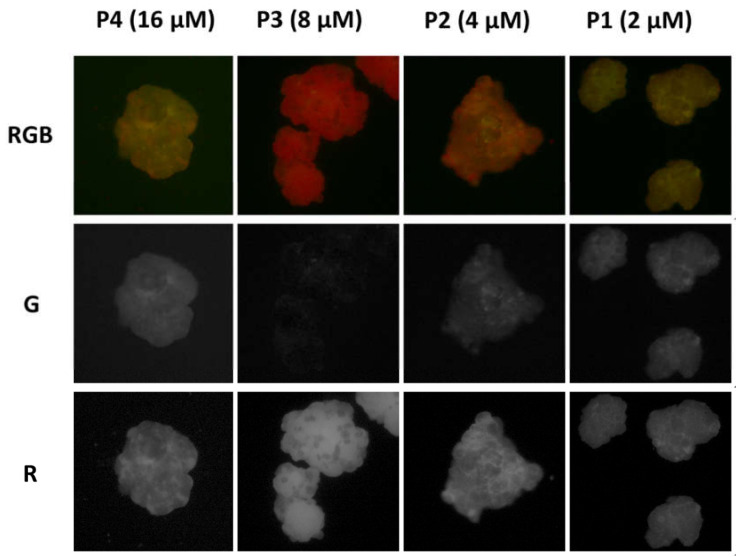
(**Top**) RGB color images of PVC particles after 2 h incubation with P in buffered water solution (pH = 7) at decreasing concentration (16, 8, 4 and 2 µM, solutions P4-P1) obtained with a fluorescence inverted microscope upon illumination with a blue LED diode in epifluorescence mode. The images were acquired with a conventional CMOS camera directly in the P solutions, without any washing. The later size of each image is 450 µm. The images were split into the three color components R, G and B with the software Image J. The G components are shown in the second line of images. The R component are shown at the (**bottom**).

Afterwards, the PVC particles were observed with an inverted fluorescence microscope under excitation at 460 nm, detecting the fluorescence with a conventional color camera after filtering with a cut-off (50% transmittance at 510 nm). The fluorescence images illustrated in Figure 5 show that PVC becomes strongly fluorescent after incubation with P1–P4 solutions and that the intensities and fluorescent colors were, to some extent, concentration dependent. In particular, the total average intensity (I_RGB_) progressively increased with the P concentration. In fact, when the red component is considered with respect to the green as the I_R_/I_B_ ratio, as plotted in Figure 4, it becomes clear that an optimal red staining was achieved at a concentration of 8 µM.

**Figure 6:**
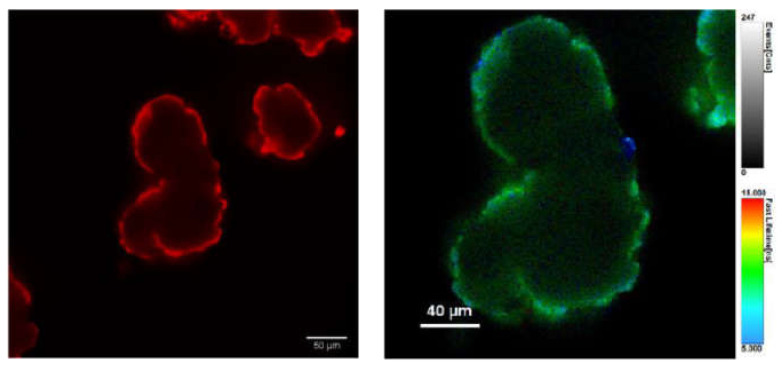
Confocal scanning fluorescence images of PVC particles after 2 h incubation with P (8 µM) in buffered water (pH = 7): (**Left**) excitation at 489 nm and detection at 595/50 nm; and (**Right**) FLIM image showing the local excited state lifetimes.

The above results indicate that indeed the adsorption of P onto the PVC surface can lead to an enhancement of the quantum yield. To confirm this hypothesis, we analyzed the red fluorescence of the stained PVC particles by confocal scanning fluorescence microscopy, measuring in particular the local excited state decay with a setup for fluorescence lifetime image microscopy (FLIM). The confocal fluorescence image upon excitation at 489 nm and red channel detection (590/50 nm), as shown in Figure 6, clearly demonstrates that the probe was absorbed only on the surface of the PVC and did not penetrate in the inner layers. On the other hand, the increase of the red emission intensity, with respect to the weak one of the P dimer in solution, is demonstrated by the FLIM image in Figure 6, where an excited state lifetime as long as 10 ns was measured for the red fluorescence on the PVC surface. Considering all these results, and the following relevant known properties of PDI derivatives, we propose for the staining process the mechanism schematized in Figure 1.

For PDI in general, it has been reported that strong π–π interactions, such as those in the P_2_ dimer, lead to a strong quenching of the typical red fluorescence with a shortening of the excited state lifetime [38]. On the contrary, less compact structures, where the overlap of the π system is less efficient (e.g., those formed in the case of the presence of bulky substituents on the aromatic PDI systems), are more intensely fluorescent and present a much longer excited state lifetime [43]. Regarding the present approach, the efficient formation of the P_2_ dimer clearly demonstrates that the π–π stacking interaction (indicated as 1 in Figure 1) prevails over the electrostatic repulsion between the terminal protonated amino groups (indicated as 2 in Figure 1). The previous is possible because the flexible ethylenedioxi-chains can easily bend to increase the distance between the positive charges and, hence, minimize the repulsion. On the contrary, when P is adsorbed on PVC, the PDI unit comes into contact with the polymer surface and the hydrophilic positive ammonium charges are directed toward the solvent creating an accumulation of positive charges on the surface. This results in the repulsion of other protonated P molecule in the solution and a reduced stacking of P on the PVC surface. As a consequence, the less aggregated PDI structures on the PVC surface present more intense fluorescence. Thus, the proposed mechanism is clearly the result of a delicate balance between attractive and repulsive forces that are different in the solution with respect to the PVC surface.

**Figure 7:**
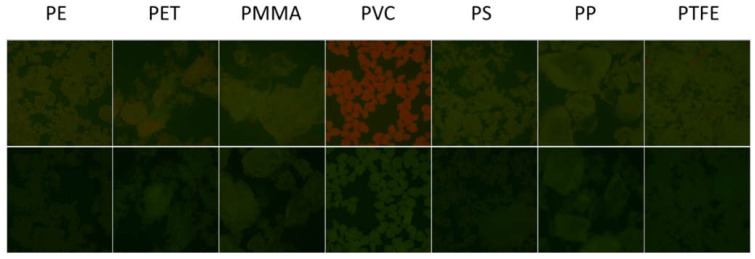
(**Top**) From left to right: RGB color images of PE, PET, PMMA, PVC, PS, PP and PTFE particles after 2 h incubation with P in buffered water solution (pH = 7) at concentration 8 µM obtained with a fluorescence inverted microscope upon illumination with a blue LED diode in epifluorescence mode. Images were acquired with a conventional CMOS camera directly in the P solutions, without any washing. The lateral size of each image is 1.8 mm. (**Bottom**) Images of the fluorescence of the same polymers in the same conditions without staining.

**Figure 8:**
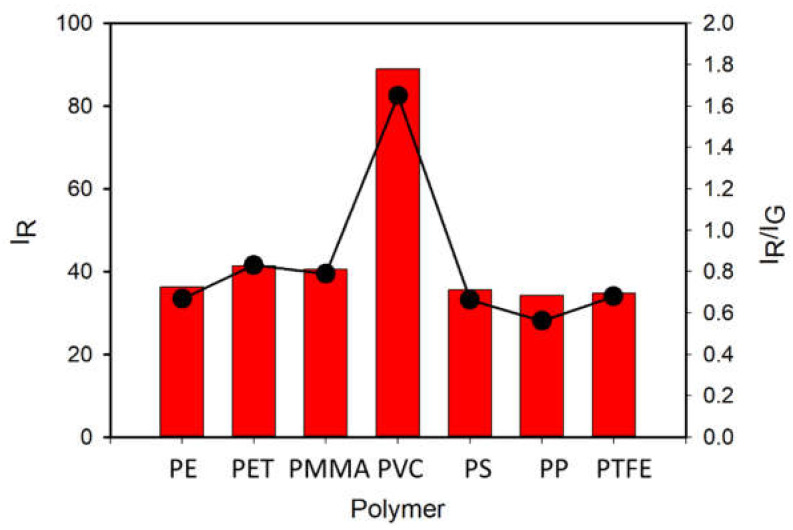
Black dots show the intensity on the red channel (I_R_) of the fluorescence images of PE, PET, PMMA, PVC, PS, PP and PTFE particles after 2 h incubation with P in buffered water solution (pH = 7) at concentration 8 µM obtained with a fluorescence inverted microscope upon illumination with a blue LED diode in epifluorescence mode. Images were acquired with a conventional CMOS camera directly in the P solutions, without any washing. The ratio of the intensities on the red and green channels is plotted as red bars.

To test the actual selectivity of P towards PVC, we compared the fluorescence of a set of polymers (5 mg) after 2 h incubation with P in buffered solution at the optimal concentration of c = 8 µM. Seven widely used polymer MP were analyzed: PVC, PE, PET, PP, PS, PMMA and PTFE. Figure 7 (top) shows the images of the different polymeric particles after 2 h of incubation with the probe using the inverted fluorescence microscope upon irradiation with blue light. The images were acquired directly in the P solution without any washing. As shown in Figure 7 (bottom), all polymers exhibited a weak green fluorescence when observed in these excitation conditions without the probe. Importantly, after incubation with the probe P, only PVC presents a peculiar red emission, while only minor changes were observed for the other polymers. The bright red color generated in the case of PVC allowed the easy detection and identification of this kind of MP. This different response can be easily observed by analyzing the intensity on the red channel or by representing the red to green intensity ratio I_R_/I_G_, as shown in Figure 8. Overall, the possibility of using P as probe for PVC MP detection possesses the typical advantages of ratiometric detection, making the identification of the target largely independent of the experimental conditions (e.g., excitation source intensity). Finally, the effects of a series of water-soluble polymers at concentration 2.5 mg/mL on the fluorescence properties of P were investigated. Poly(acrylic) acid, poly(styrene sulfonic acid sodium salt) and poly(vinyl alcohol) were tested. In all cases, a very minor change in the fluorescence intensity was measured with a final quantum yield that was lower than 0.1% for all the aforementioned polymers. Finally, polyaniline, a less common water-insoluble polymer, was tested, where no fluorescence from the polymeric particles was observed after 2 h incubation with P (c = 8 µM).

It should be stressed that the method we present is very effective for the qualitative identification of PVC MP, but, in the present form, it does not allow a precise quantification. The observed selectivity for PVC was in part expected considering the general good solubility of PDI derivatives in chlorinated solvents such as DCM and dichloroethane that reveals a good affinity for chlorinated species and favors the disaggregation of PDI units [40]. As discussed in recent papers, the interaction between organic molecules and MP is the result of several interactions among which the halogen–aromatic interaction can be concluded to be the one playing a major role in the case of PVC–PDI interaction [44,45].

## 4. Conclusions

We demonstrated a new approach, established on the design of a PDI-based probe, for the direct labeling and recognition of MP in water (just by mixing). As an advantage, this PDI molecule behaves as a fluorogenic probe, being non-fluorescent in water at neutral pH but becoming intensely red fluorescent upon adsorption on PVC MP. We demonstrated that the labeling process presents great advantages: it can be performed at room temperature and in a short time (2 h). Moreover, the fluorescence of the MP can be detected without any further treatment of the sample (no-washing). The probe exhibited an intense green fluorescence in organic solvent that, in water at neutral pH = 7.0, is almost completely quenched because of the formation of dimers, giving very weak background signal. Interestingly, the probe showed considerable selectivity to PVC particles, which presented an intense red emission after staining that could be detected, under excitation with a blue diode, with a conventional CMOS color camera directly in the probe solution. It is worth noting that the probe was prepared through an environmentally friendly, fast, versatile, cheap and easily scalable method based on microwave heating.

These results pave the way to the development of new specific, fast and convenient probes for the detection of MP in real samples with a simple mixing and no-washing approach using widespread electronic devices.

## Figures and Tables

**Table 1:** Photophysical properties of P in water (pH = 7) at different concentrations.

C (µM)	ε (M^−1^cm^−1^) 500 nm	Φ_1_ % λ_exc_ = 470 nm	τ_1_ (ns) λ_exc_ = 405 nm λ_em_ = 540 nm	r_1_ λ_exc_ = 470 nm	Φ_2_ % λ_exc_ = 590 nm	τ_2_ (ns) λ_exc_ = 635 nm λ_em_ = 650 nm	r_2_ λ_exc_ = 590 nm
2 (P1)	37,000	1.6	4.47	0.026	0.03	<0.1	0.25
4 (P2)	38,000	1.1	4.54	0.023	0.02	<0.1	0.26
6 (P3)	33,000	0.6	4.56	0.023	0.03	<0.1	0.28
16 (P4)	34,000	0.4	4.58	0.024	0.05	<0.1	0.27

## Data Availability

The data presented in this study are contained within the article.
